# Altered Signaling Pathways Revealed by Comprehensive Genomic Profiling in Patients With Unknown Primary Tumors

**DOI:** 10.3389/fonc.2022.753311

**Published:** 2022-03-24

**Authors:** Zhen Yang, Wei Cui, Ruoying Yu, Xinhua Dong, Jian Zhao, Lu Dai, Qiuxiang Ou, Hua Bao, Xue Wu, Chuanxin Wu, Jinhuo Lai

**Affiliations:** ^1^ Department of Gastrointestinal Surgery, First Affiliated Hospital of Zhengzhou University, Zhengzhou, China; ^2^ Department of Colorectal Surgery, Ningbo Medical Center Lihuili Hospital, Ningbo, China; ^3^ Translational Medicine Research Institute, Geneseeq Technology Inc., Toronto, Canada; ^4^ Department of Thoracic Surgery, Affiliated Cancer Hospital and Institute of Guangzhou Medical University, Guangzhou, China; ^5^ Department of Medical Oncology, Fujian Medical University Union Hospital, Fuzhou, China

**Keywords:** carcinoma unknown primary, DNA damage repair, targeted therapy, homologous recombinant repair, clinically actionable mutation

## Abstract

**Purpose:**

Carcinoma of unknown primary (CUP) is a clinically aggressive disorder with early tumor dissemination. Identifying molecular traits of CUP can be not only beneficial for a better therapeutic approach but also potentially valuable for patients with general metastatic dissemination.

**Patients and Methods:**

We retrospectively investigated a total of 35 unique CUP cases. Tumor tissue samples were available in 26 patients, and plasma samples were available in 22 patients. Targeted sequencing was performed with a panel of 416 pan cancer-related genes.

**Results:**

A genomic landscape of the CUP cohort showed that *TP53* mutation was the most frequently observed mutation while *MYC* amplification was the most common CNV. Aberrant TP53, RTK-RAS, and PI3K signaling pathways were also prevalent, identified in more than half of the cases with tumor tissue. Around 58% of the CUP cases harbored homologous recombinant repair (HRR) pathway gene alterations. The tumor mutational load of CUP patients with altered HRR pathway displayed a significant increase than that of patients with intact HRR. Clinically actionable mutations were identified in eight patients, which may benefit from targeted therapies. Eight patients were treated with platinum-based chemotherapy, showing different responses, HRR, and LOH status.

**Conclusion:**

Collectively, our data have provided much-need insights into the treatment options for patients diagnosed with CUP in the era of precision medicine.

## Introduction

Carcinomas of unknown primary (CUP) which account for 3%–5% of all human cancers are heterogeneous neoplasms which remain a diagnostic and therapeutic challenge for physicians. CUP patients usually present with histologically confirmed metastatic cancer but unidentifiable primary tumor location (1, 2). Adenocarcinoma (ADC) is the most common type of CUP which contributes up to 60% of total CUP cases. Other less common histological types are undifferentiated carcinomas (30%), squamous cell carcinoma (SCC, 5%–8%), and neuroendocrine carcinoma (2%–4%) (3).

Due to the late tumor presentation and difficulty in diagnosis, the prognosis of CUP is poor. The median survival of the non-selective platinum-based combination chemotherapy is around 6 to 8 months with a response rate of 20% to 40% (4, 5). Target therapy has been given in some cases with a promising response. In one study of 60 CUP patients, carboplatin and paclitaxel were given as first-line treatment and bevacizumab plus erlotinib as maintenance treatment. A response rate of 53% and overall survival of 13 months were reached (6). In another study of 47 patients, bevacizumab plus erlotinib was administrated as second-line therapy with a response rate of 10% and median survival of 7.4 months (7).

Comprehensive genomic profiling has been performed in previous studies to get a better understanding of the molecular features of CUP patients. Gene alterations in *TP53*, *KRAS*, *MYC*, *ARID1A*, and *PIK3CA* have been frequently observed with clinically relevant alterations in the receptor tyrosine kinase (PTK)/RAS signaling pathway in different Western CUP cohorts (2, 8). In this retrospective study, targeted gene sequencing was used to evaluate tumor tissue or plasma from 35 CUP patients across China, which provided more insights into treatment options for this malignant disease.

## Method

### Patients and Sample Collection

As shown in **Supplementary Figure 1**, a total of 37 patients clinically diagnosed as carcinoma of the unknown primary were retrospectively enrolled from multiple hospitals across China according to the NCCN guidelines. The carcinoma of unknown primary was defined as a histologically proven metastatic malignant tumor whose primary site cannot be identified during pretreatment evaluation. Patient follow-up has also been performed to confirm that the primary tumors remained unknown until the time of this study. Two patients were excluded due to the lack of evaluable tumor tissue and plasma or the difference in the targeted sequencing panel used. Finally, 26 tumor tissue and 22 plasma samples from a total of 35 CUP patients were subjected for further analysis. The tumor and plasma samples were all collected after treatment. Written consent was collected from each patient. The NGS tests were performed in a centralized clinical testing center (Nanjing Geneseeq Technology Inc.) with a target gene panel of 416 genes according to protocols reviewed and approved by the ethical committee of each participating hospital. 5 to 10 ml of peripheral blood was collected from each patient in EDTA-coated tubes (BD Biosciences, San Jose, CA, USA). Plasma was extracted within 2 h of blood collection and shipped to the central testing laboratory within 48 h. Formalin-fixed paraffin-embedded (FFPE) tumor tissue blocks/sections or fresh tumor tissues were obtained from the hospitals, and with confirmation by the pathologists from the centralized clinical testing center for diagnosis and tumor purity.

### DNA Extraction and Quantification, Library Preparation

The DNA extraction, quantification, and library preparation were performed as previously described (9). In brief, FFPE samples were de-paraffinized with xylene, and DNA was extracted using the QIAamp DNA FFPE Tissue Kit (Qiagen, Hilden, Germany) according to the manufacturer’s protocols. Genomic DNA from fresh tumor tissue was extracted using the DNeasy Blood & Tissue Kit (Qiagen) according to the manufacturer’s protocols. Peripheral blood samples were centrifuged at 1,800g for 10 min. Then, the plasma was isolated for extraction of cfDNA and the genomic DNA of white blood cells in sediments served as normal control. The Circulating Nucleic Acid Kit (Qiagen, Germany) was used to purify cfDNA from plasma. The genomic DNA from white blood cells was extracted using DNeasy Blood and Tissue Kit (Qiagen). Genomic DNA was qualified using a NanoDrop 2000 (Thermo Fisher Scientific, Waltham, MA), and cfDNA fragment distribution was analyzed on a 2100 Bioanalyzer using the High Sensitivity DNA Kit (Agilent Technologies, Santa Clara, CA). All DNA was quantified using the dsDNA HS assay kit on a Qubit 3.0 fluorometer (Life Technologies, Carlsbad, CA, USA) according to the manufacturer’s recommendations. Sequencing libraries were prepared using the KAPA HyperPrep Kit (KAPA Biosystems, Woburn, MA, USA) with an optimized manufacturer’s protocol and sequenced as previously described (9). The mean sequencing depths of tumor tissue and plasma were 500× and 3,000×, respectively.

### Data Processing

Sequencing data were processed as previously described (9). In brief, the data were first demultiplexed and subjected to FASTQ file quality control to remove low-quality data or N bases. Qualified reads were mapped to the reference human genome hg19 using the Burrows–Wheeler Aligner, and the Genome Analysis Toolkit (GATK 3.4.0) was employed to apply the local realignment around indels and base quality score recalibration. Picard was used to remove PCR duplicates. VarScan2 was employed for the detection of single-nucleotide variations (SNVs) and insertion/deletion mutations. SNVs were filtered out if the mutant allele frequency (MAF) was less than 1% for tumor tissue and 0.3% for plasma samples. Common SNVs were excluded if they were present in >1% of the population in the 1000 Genomes Project or the Exome Aggregation Consortium (ExAC) 65,000 exomes database. The resulting mutation list was further filtered by an in-house list of recurrent artifacts based on a normal pool of whole blood samples. Parallel sequencing of matched white blood cells from each patient was performed to further remove sequencing artifacts, germline variants, and clonal hematopoiesis. The copy number alterations were analyzed as previously described (10, 11). The tumor purities were first estimated using ABSOLUTE (12). Somatic CN alteration events were assigned based on sample-ploidy values calculated in the FACETS algorithm (13). Loss of heterozygosity (LOH) was also calculated using FACETS and determined using the minor copy number estimates of each segment for genes in the targeted panel. The minor copy number is by definition 0 in an LOH event (14, 15). Structural variants were detected using FACTERA with default parameters (16). The fusion reads were further manually reviewed and confirmed on Integrative Genomics Viewer (IGV).

The microsatellite instability (MSI) was determined by evaluating 52 embedded mononucleotide repeats with a minimum of 15-bp repeats that were included in the sequencing panel. The baseline length distribution of each repeat was determined from a pool of microsatellite-stable samples. A sample was identified as MSI if more than 40% of the qualified sites displayed instability. Tumor mutational burden (TMB, mutation per megabase) was determined based on the number of somatic base substitutions and indels in the targeted regions of the gene panel covering 0.85 Mb of coding genomes, excluding known driver mutations as they are overrepresented in the panel. Samples within the highest mutation-load tertile (top 33.3%) were classified as having high TMB, which was equal to ≥20 mutations/Mb in TMB, as previously described (17). LOH-high was also defined as the samples within the highest LOH tertile (top 33.3%), which was equal to ≥33% in LOH.

Homologous recombination (HR) pathway genes were defined according to previous publications including the genomic analyses of ovarian carcinoma published by the Cancer Genome Atlas Research Network (TCGA) and clinical trials of the PARP inhibitor in different cancer types (NCT04042831, NCT03207347, and NCT03209401) (18–21). The HRR genes included *BRCA*1/2, *PALB2*, *CHEK2*, *ARID1A*, *NBN*, *BARD1*, *BRIP1*, *RAD50*, *RAD51C*, *RAD51D*, *RAD54L*, *MRE11*, the set of Fanconi anemia genes (*FANCA*, *FANCC*, *FANCD2*, *FANCE*, *FANCF*, *FANCG*, *FANCL*, FANCM), *C11orf30 (EMSY)*, the DNA damage sensing genes *ATM* and *ATR*, and *PTEN*.

## Results

### Patient Cohort

This CUP cohort included 24 males (69%) and 11 females (31%) with a median age at diagnosis of 62 years, ranging from 42 to 82 years. Of the 35 CUP cases included in this retrospective study, 42% (11 out of 35) was adenocarcinoma (ACUP) and 23% (8 out of 35) was squamous cell cancer (SCUP). Two cases were identified as poorly differentiated CUP. Histology type was unknown in 40% (14 out of 35) of cases. The distribution of tumor location is shown in **Table 1**, and the detailed tumor metastasis status is shown in **Supplementary Table 1**. The numbers of organ metastatic lesions were classified as follows: lymph node metastasis (10, 29%), single-organ metastasis (8, 23%), organ and lymph node metastasis (3, 8%), multiple-organ metastasis (3, 8%), and unknown metastasis (11, 31%). In patients with single-organ metastasis, tumor was found in the lung (2), liver (2), bone (2), retina (1), and abdomen (1). In patients with organ and lymph node metastasis, organ metastasis was detected in the bone, tongue, bone, and adrenal gland, respectively. In the three patients with multiple organ metastasis, two patients with both lung and bone metastasis showed metastasis in intestine and gall bladder respectively. The other patient had metastasis in the liver and gut.

**Table 1:** Clinical information of CUP patients.

Characteristics	CUP cohort n = 35
**Sex no. (ratio)**	
Male	24 (69%)
Female	11 (31%)
**Age**	
Range	42–82 years old
Average	62 years old
**Histology no. (ratio)**	
ADC	11 (42%)
SCC	8 (23%)
Poorly differentiated	2 (5%)
Unclassifiable	14 (40%)
**Anatomical location no. (ratio)**	
Lymph node only	10 (29%)
Single-organ metastasis	8 (23%)
Organ and lymph node metastasis	3 (8%)
Multiple-organ metastasis	3 (8%)
Unknown	11 (31%)
**Family history of cancer no. (ratio)**	
Esophageal cancer	3 (8%)
Lung cancer	2 (6%)
Colon cancer	2 (6%)
Pancreatic cancer	1 (3%)
Cervical cancer	1 (3%)
Ampullary cancer	1 (3%)
Unknown cancer	1 (3%)
No family history	22 (62%)
Unknown	2 (6%)

ADC, adenocarcinoma; SCC, squamous cell carcinoma.

CUP has been shown associated with the occurrence of lung, kidney, and colorectal cancers in families (22). Here eleven patients had cancer occurrence in the family, including three families with esophageal cancers, two families with lung cancers, and two with colon cancers (**Table 1** and **Supplementary Table 1**). Both the sister and brother of patient 30 were diagnosed with lung cancer while the brother of patient 15 was diagnosed with lung cancer. The brother of patient 10 and the mother of patient 07 were diagnosed with colon cancer. However, we did not find any potentially deleterious germline variant that should have prompted patients to undergo germline testing nor had they had undergone germline testing before the study.

### Genomic Landscape of Patients With Unknown Primary Tumor

**Figure 1:**
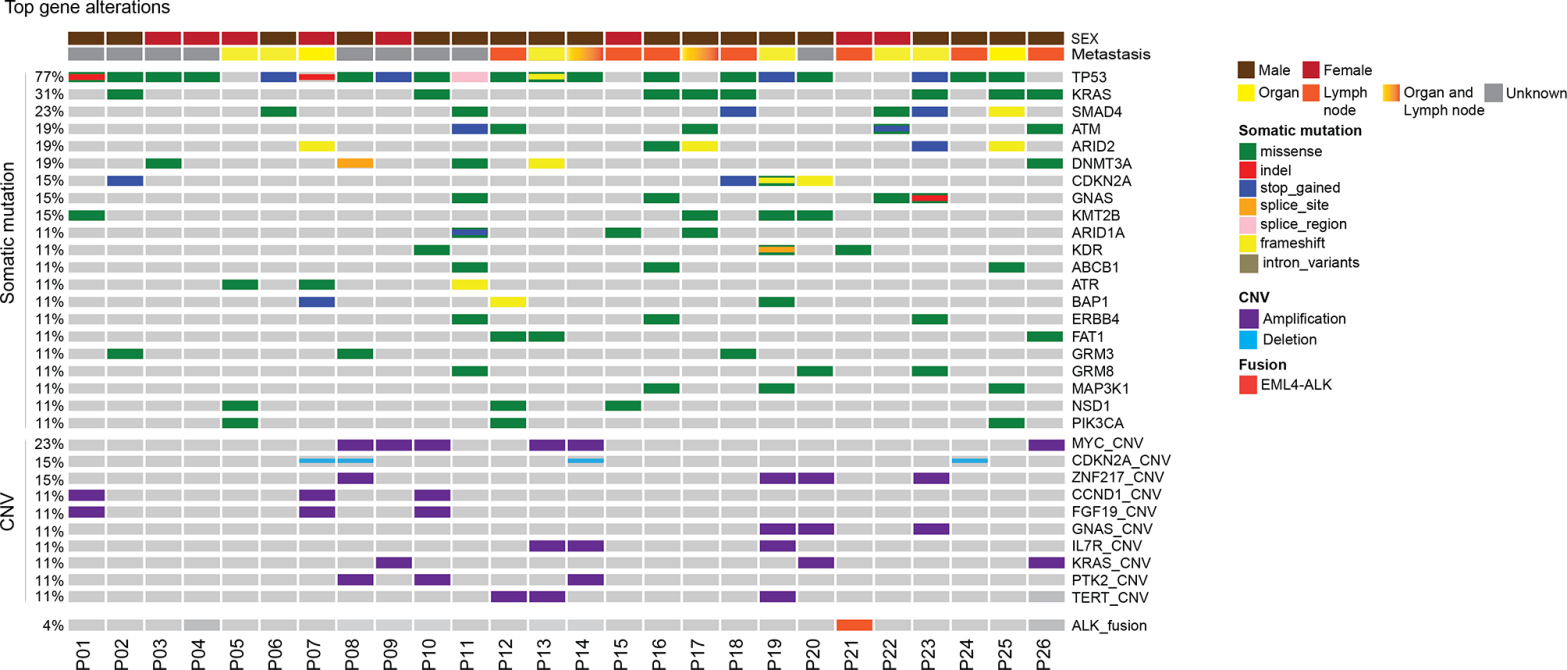
Mutational landscape of patients with unknown primary tumor. Distribution of individual gene mutations and copy number variation, *ALK* fusion in the cohort as assessed by target panel sequencing. The sex of patients was provided as bars on the top. The mutation types were indicated by the color on the right. Each column represents one patient.

In this CUP cohort, tissue samples were available in 26 patients and plasma samples were available in 22 patients. Tumor tissues were from metastatic tumors including lymph nodes tumors (13), lung tumors (4), liver tumors (2), bone (2), retina (1), and abdomen (1). The genomic landscape of CUP patients in tumor tissue and plasma is shown in **Figure 1** and **Supplementary Figure 2**, respectively. The detailed mutation list is shown in **Supplementary Table 2**. Commonly altered genes found in both tissue and plasma samples included *TP53* (77% of tissue, 64% of plasma), *KRAS* (31% of tissue, 36% of plasma), and *SMAD4* (23% of tissue, 14% of plasma), similar to reported CUP cohorts (23). *MYC* amplification was the most frequently observed copy number variation (CNV) which accounted for 23% (6 out of 26) of total cases. Other frequently observed CNVs included *CDKN2A* deletion (15%) and *ZNF217* amplification (15%). *EML4-ALK* fusion was detected in one patient.

### Pathway Analysis of Patients With Unknown Primary Tumor

**Figure 2:**
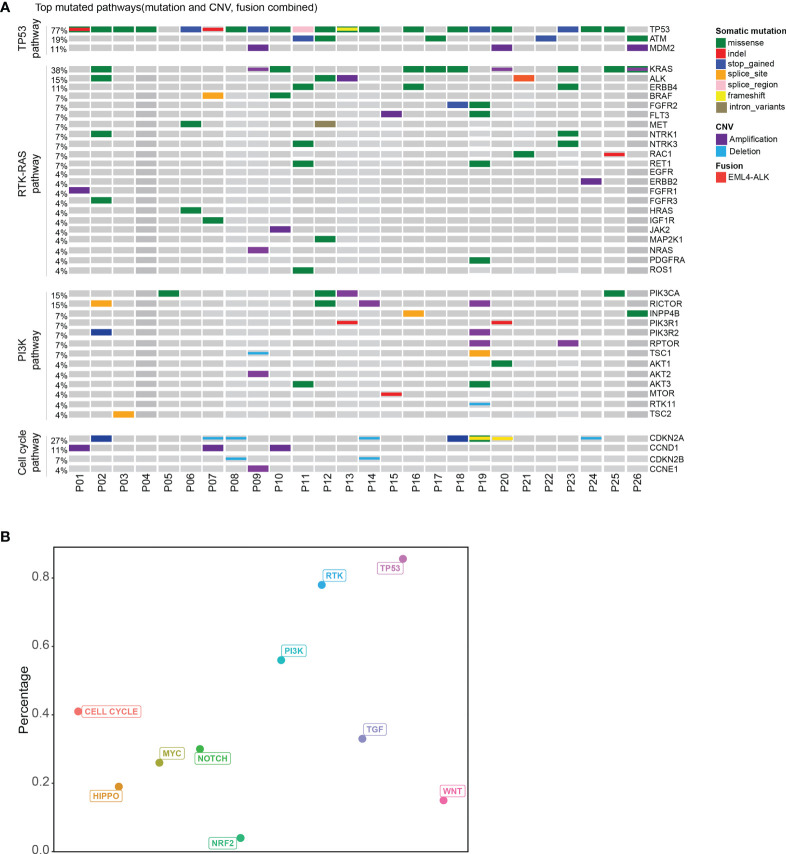
Pathway analysis of patients with unknown primary tumor. **(A)** Top mutated canonical pathways in the CUP cohort. The mutation types were indicated by the color on the right. Each column represents one patient. **(B)** The percentage of patients with different altered canonical pathways. Distribution of patients with mutated HRR gene.

**Figure 3:**
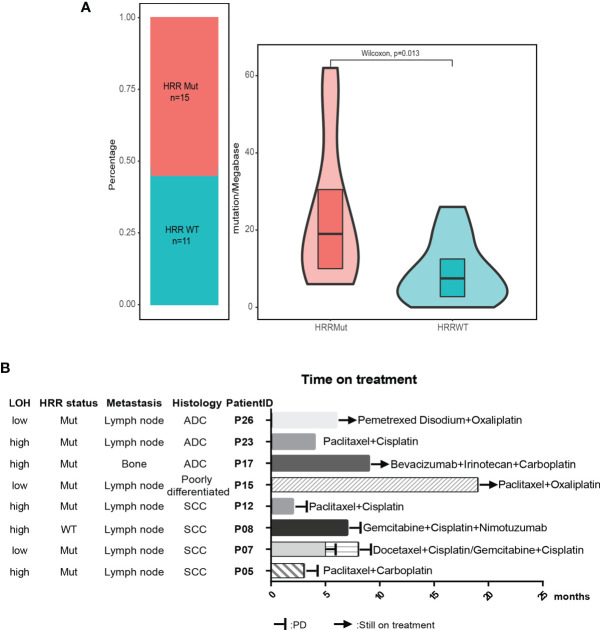
HRR pathway alteration status in patients with unknown primary tumor. **(A)** Patients with mutated HRR had a higher TMB compared with patients with wild-type HRR. HRR, homologous recombination repair; WT, wild type; Mut, mutated. **(B)** Available treatment history of patients with unknown primary tumor. Swimmer’s plot of time on treatment for eight patients treated with different combination chemotherapy. Patients 5, 7, 8, and 12 had documented PD. Patients 15, 17, and 26 were still on treatment. The information of drug response in patient 23 was unknown. ADC, adenocarcinoma; SCC, squamous cell carcinoma.

Based on the ten canonical pathways profiled by The Cancer Genome Atlas (TCGA) (24), we looked into the frequently altered pathways in CUP. As shown in **Figure 2**, the TP53 signaling pathway (23/26, 88%) was the most frequently altered canonical pathway in CUP. RTK-RAS (20/26, 77%) and PI3K (15/26, 58%) signaling pathways were also found to be in tumor tissues of more than half of CUP patients. Intriguingly, 58% (15/26) of the CUP cases harbored alterations in homologous recombination pathways (**Figure 3** and **Supplementary Table 3**).

We further compared the altered pathways in CUP with three published cancer cohorts (one stage II–IV esophageal squamous cell cancer cohort/ESCC, one metastatic lung cancer cohort, one stage II–III colorectal cancer cohort) (25–27) (**Supplementary Figure 3**). The tumor tissues of the ESCC and lung cancer cohorts were sequenced with the same 416 gene panels while the colorectal cancer cohort was sequenced with a 425-gene panel covering the 416 genes. Lung cancer and colorectal cancer have been reported with a high ratio of RTK-RAS pathway gene alterations while the ESCC cohort and colorectal cancer have a high ratio of TP53 pathway alterations according to other publications (28–31). The HRR pathway has been reported to be enriched in esophageal squamous cell cancer (32). In this study, TP53 and RTK-RAS pathways were the top two pathways altered in CUP. The ratio of altered *RTK-RAS* in lung cancer was significantly higher than that in ESCC and colorectal cancer (p < 0.001). The difference in the RTK-RAS ratio between CUP and lung cancer was insignificant due to the limited cohort size. The ratio of TP53 pathway alterations was insignificant among the four groups but with a trend of decrease in lung cancer. Interestingly, the CUP cohort showed a higher ratio of patients with HRR pathway gene mutations than the other three cohorts (p < 0.05).

The median tumor mutational burden (TMB) of the 26 CUP cases with tumor tissue was 12.5 mutation/Mb. The differences in TMB were significant between patients with HRR wild-type and mutated HRR (p = 0.013, mean TMB HRR WT vs. mutated: 9.9 vs. 23.9 mutation/Mb) **Figure 2B**). The microsatellite instability (MSI) analysis revealed that the 26 tumor specimens were all microsatellite stable (MSS). Genomic loss of heterozygosity (LOH) was also assessed, showing a median LOH of 28.0%. Although there was no significant difference in the LOH status between HRR WT and mutated tumors, 67% (10/15) of HRR-mutated tumors displayed a LOH higher than 20% and 40% (6/15) had a LOH over 33% (**Supplementary Table 3**).

### Clinically Actionable Mutations in Patients With Unknown Primary Tumor

According to the OncoKB database, clinically actionable mutations identified in this cohort were *KRAS* G12C(3), *PIK3CA* E545K(2), *PIK3CA* E542K(1), *BRAF* V600L(1), *EML4*-*ALK*(1), and *ALK* L1196M(1) (**Table 2**), accounting for 23% (8 out of 35) of total cases. 78% of the actionable mutations were detected in both plasma and tissue samples from the same patient. The available treatment history of patients is shown in **Figure 3B**. Platinum-based chemotherapy was applied in eight cases without intervention of tyrosine kinase inhibitor (TKI) therapy.

**Table 2:** Actionable mutations detected in patients with unknown primary tumor.

Actionable mutation	Possible drug application	Patient ID	Histology	Allele frequency	Reference
*KRAS* G12C	AMG-510	P02	SCC	Plasma 23.33%; Tissue 4.26%	(Chakravarty et al., 33)
		P16	NA	Plasma 1.08%; Tissue 1.08%	
		P26	ADC	Plasma 58.96%; Tissue 77.75%	
*PIK3CA* E545K	Buparlisib, buparlisib + fulvestrant, copanlisib, GDC-0077, serabelisib, taselisib, taselisib + fulvestrant	P05	SCC	Plasma 14.27%; Tissue 39.25%	
		P12	SCC	Plasma 3.32%; Tissue 3.32%	
*PIK3CA* E542K		P25	SCC	Plasma 5.44%; Tissue 13.55%	
*BRAF* V600L	Vemurafenib	P10	NA	Tissue 0.7%	
*EML4-ALK* fusion	Alectinib, ceritinib, crizotinib	P21	ADC	Tissue 18.61%	
*ALK* L1196M	Crizotinib	P12	SCC	Plasma 0.32%; Tissue 0.32%	
TMB-high	Immunotherapy inhibitors	P02, P12, P13, P16, P17, P20, P22, P24, P26	–	–	(Marabelle et al., 34)
LOH-high, HRR mutated	PARP inhibitors	P05, P10, P12, P16, P17, P23	–	–	(Coleman et al., 35)

The actionable mutations were defined according to the OncoKB database. Level 3A, compelling clinical evidence; Level 1, FDA-approved; Level R2, resistance-compelling clinical evidence.

NA, not available.

Four patients with metastatic squamous cell cancer (SCC) in lymph nodes (P05, P07, P08, P12) progressed on platinum-based chemotherapy within 10 months of treatment (**Figure 3B**). Although three of four patients displayed HRR mutated and LOH-high, the PARP inhibitor may not be a good option for these patients. Meanwhile, *PIK3CA* E545K mutation was identified in tumor tissue and plasma of P05 and 12, and no other known driver mutation was identified in both cases (**Table 2**). Target therapy with PI3K inhibitors such as buparlisib, copanlisib, and taselisib might be a better treatment option, especially for patient 05 with an allele frequency (AF) of 39.25% detected in tissue. Patient 26 with metastatic adenocarcinoma in lymph node was treated with pemetrexed disodium plus oxaliplatin for 6 months and achieved stable disease (SD). *KRAS* G12C was found at an AF of 77.75% in tissue and 58.96% in plasma, indicating that the *KRAS* G12C inhibitor such as AMG-510 could be beneficial for this patient. P17 and P23 were HRR-mutated and LOH-high adenocarcinoma. Carboplatin or oxaliplatin-based chemotherapy was administrated for more than 5 months. PARP inhibitors may be used for these patients if they reach a platinum-free interval of 6 months or longer.

## Discussion

In this retrospective study, we explored the molecular features of Chinese patients with unknown primary tumors using next-generation sequencing with a multigene panel. The genomic landscape of Chinese CUP patients was similar to the Western population with high frequent alterations in *TP53* and *KRAS.* Interestingly, the incidence of *SMAD4* alteration (23% in tissue, 14% in plasma) was higher in the Chinese population compared to the Western cohort (6%) (8). The enrichment of TP53, RTK-RAS, and PI3K signaling pathways indicated the possibility of targeted therapies such as RTK and PI3K inhibitors in CUP patients. The PI3K inhibitor has been administrated in a SCUP patient and achieved the best response of stable disease with a PFS of 6.5 months (36). Other targeted drugs such as crizotinib and gefitinib have also been used in CUP patients with promising outcomes (37).

Poly(adenosine diphosphate-ribose) polymerase (PARP) inhibitor, such as olaparib and niraparib, can trap PARP on DNA at sites of single-strand breaks, therefore preventing the single-strand break repair and generating double-strand breaks that cannot be repaired in tumors with homologous recombination repair defects (38). The use of olaparib provides substantial benefits among women with newly diagnosed advanced ovarian cancer and a BRCA1/2 mutation (38). Currently, the efficacy of olaparib was tested in a phase II clinical trial (CUPISCO) of patients with cancer of unknown primary site (39). Niraparib, a PARP inhibitor, has demonstrated antitumor activity in homologous recombination deficiency-positive (HRD), platinum-sensitive disease (40). The beneficial effects of niraparib were also found consistent regardless of BRCA status (41). HRD can be tested using different strategies including germline HR gene mutation screening, somatic HRR gene mutation screening, and evaluation of a genomic scar (for instance, LOH). Here over half of CUP patients had somatic HRR gene alterations and around 60% of patients had a LOH status higher than 16%. Thus, CUP patients with an altered HRR pathway might still have the opportunity to benefit from the PARP inhibitor after progressing on the conventional chemotherapy. Further studies are warranted to validate our observation and unveil the possible mechanism associated with the enrichment of HRR pathway gene alterations. Immunotherapy is another potential treatment that can be applied to CUP patients. Several phase II clinical trials have been conducted to evaluate the efficacy of pembrolizumab in CUP patients (37). It is of great interest to see whether CUP patients with increased TMB and HRR deficiency can achieve better responses to immunotherapy or PARP inhibitors.

The major limitations of this study included small cohort size and the lack of clinic-pathological risk stratification and treatment history which prevent us from further evaluating the correlation between genomic alterations and clinical outcomes. In eight patients with available chemotherapy history, all plasma and tumor tissue samples were posttreatment samples. Thus, we were not able to stratify samples into before- or after-treatment groups which might have helped us to get into correlations between gene alterations with chemotherapy. Limited information provided by panel sequencing compared to whole-exome/genome sequencing was another limitation, which might also have had an impact on the genomic LOH calculation. Further study is warranted to validate our observation and evaluate the efficiency of target therapy and immunotherapy in CUP cohort besides the standard chemotherapy.

## Data Availability Statement

The original contributions presented in the study are publicly available. This data can be found here: https://ngdc.cncb.ac.cn/gsa/, HRA002101.

## Ethics Statement

The studies involving human participants were reviewed and approved by First Affiliated Hospital of Zhengzhou University. The patients provided written informed consent to participate in this study.

## Author Contributions

Study design: JL, CW, ZY, WC, RY, XW. Data acquisition: ZY, WC, RY, XD, JZ, LD. Data analysis: ZY, WC, RY, HB, LD. Manuscript writing: ZY, WC, RY, QO, JZ. Study supervision: JL, CW, XW. All authors contributed to the article and approved the submitted version.

## Funding

This study was supported by Foundation of Henan Educational Committee (14B320014, 20B320034), Fujian Science and Technology Innovation Joint Fund Project(2018Y9063), Grants from the Science and Technology Program of Guangzhou Health Commission (20211A011088).

## Conflict of Interest

RY, QO, HB, and XW are shareholders or employees of Geneseeq Technology Inc., China.

The remaining authors declare that the research was conducted in the absence of any commercial or financial relationships that could be construed as a potential conflict of interest.

## Publisher’s Note

All claims expressed in this article are solely those of the authors and do not necessarily represent those of their affiliated organizations, or those of the publisher, the editors and the reviewers. Any product that may be evaluated in this article, or claim that may be made by its manufacturer, is not guaranteed or endorsed by the publisher.

## Abbreviations

DDR, DNA damage repair; HRR, homologous recombinant repair; CUP, carcinoma unknown primary; ADC, adenocarcinoma; RTK, receptor tyrosine kinase; SNVs, single-nucleotide variations; CNVs, copy number variations; TMB, tumor mutational burden; TKI, tyrosine kinase inhibitor.
