# Supplementary material for: Altered Signaling Pathways Revealed by Comprehensive Genomic Profiling in Patients With Unknown Primary Tumors
Source: Front Oncol. 2022 Mar 24;12:753311. doi: 10.3389/fonc.2022.753311 (PMC8991684; doi:10.3389/fonc.2022.753311)

**Supplementary Figure 1. The patient enrollment process of the studied cohort.**

**
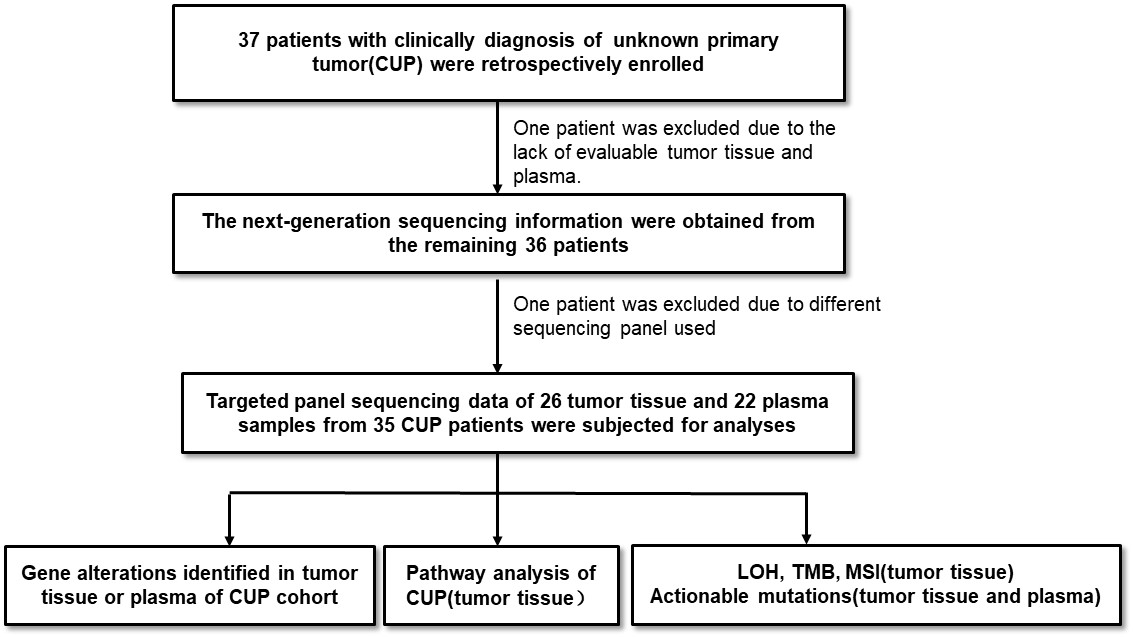
**

**Supplementary Figure 2. Mutation plot of patients with unknown primary using somatic mutations detected in plasma samples.** Top mutated genes were shown. The gene alteration types were indicated by the color on the right.


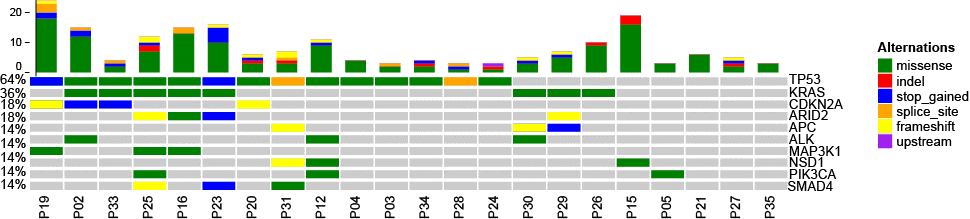


**Supplementary Figure 3. The ratio of CUP, ESCC, lung cancer, colorectal cancer patients with different pathway gene alterations.** (A) the ratio of CUP, ESCC, lung cancer, colorectal cancer patients with TP53 and RTK-RAS pathway gene alterations. (B) the ratio of CUP, ESCC, lung cancer, colorectal cancer patients with HRR pathway gene alterations. WT: wild type; MUT: mutated ESCC: Esophageal squamous cell cancer. ns: not significant; ***: p˂0.001; * p˂0.05. p value was calculated using Fisher’s exact test.


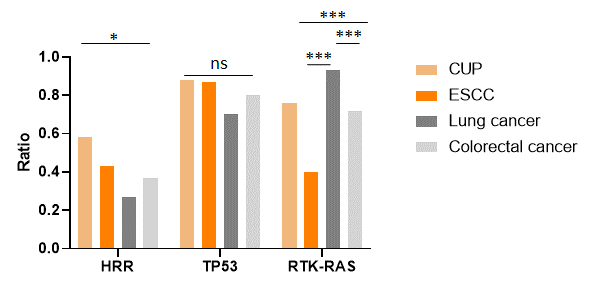

Supplement: Supplementary file 1 [file DataSheet_1.docx]
