# Supplementary material for: Altered Signaling Pathways Revealed by Comprehensive Genomic Profiling in Patients With Unknown Primary Tumors
Source: Front Oncol. 2022 Mar 24;12:753311. doi: 10.3389/fonc.2022.753311 (PMC8991684; doi:10.3389/fonc.2022.753311)
Supplement: Supplementary file 2 [file DataSheet_2.docx]

**Supplementary Table 1. Summary of patients with unknown primary tumor**

| **Patient ID** | **Age range at Diagnosis** | **Metastasis** | **Family history** |
| --- | --- | --- | --- |
| P01 | 60-65 | Lymph node; Bone | No family history |
| P02 | 60-65 | Lymph node | No family history |
| P03 | 66-70 | Retina | No family history |
| P04 | 55-60 | Lymph node | No family history |
| P05 | 60-65 | Lymph node | No family history |
| P06 | 50-55 | Lymph node | Father and mother had esophageal cancer |
| P07 | 60-65 | Lymph node | Mother had colon cancer |
| P08 | 55-60 | Lymph node | Father had pancreatic cancer |
| P09 | 55-60 | NA | No family history |
| P10 | 66-70 | Lymph node;Bone; Adrenal gland | Brother had colon cancer |
| P11 | 60-65 | Bone | No family history |
| P12 | 66-70 | Lymph node | No family history |
| P13 | 75-80 | NA | Father had esophageal cancer |
| P14 | 50-55 | NA | Brother had lung cancer |
| P15 | 50-55 | Lymph node | No family history |
| P16 | 70-75 | NA | No family history |
| P17 | 70-75 | Bone | Two uncles, one aunt and one nephew had esophageal cancer |
| P18 | 80-85 | Lung | No family history |
| P19 | 50-55 | Lung | No family history |
| P20 | 80-85 | Lung; Bone; Gut | No family history |
| P21 | 50-55 | Lymph node | No family history |
| P22 | 40-45 | Abdominal | No family history |
| P23 | 50-55 | Lymph node; Tongue | No family history |
| P24 | 50-55 | Liver; Gut | No family history |
| P25 | NA | NA | Unknown |
| P26 | 66-70 | Lymph node | No family history |
| P27 | 66-70 | NA | No family history |
| P28 | 75-80 | NA | Daughter had cervical cancer |
| P29 | 60-65 | NA | Brother and sister had lung cancer |
| P30 | 60-65 | Liver | Father had ampullary cancer |
| P31 | 75-80 | Lung; Bone; Intestine | Father had cancer of unknown primary |
| P32 | 75-80 | NA | Unknown |
| P33 | 45-50 | Liver | No family history |
| P34 | 60-65 | NA | Unknown |
| P35 | 85-90 | NA | No family history |

**Supplementary Table 2. The mutation list of gene alterations detected in the tumor tissue.** AA change: amino acid change.

| **Patinet ID** | **Gene** | **Mutation type** | **AAChange** |
| --- | --- | --- | --- |
| P01 | CCND1 | Amplification |  |
| P01 | FGFR1 | Amplification |  |
| P01 | TP53 | indel | c.652_654delGTG(p.V218del) |
| P01 | TP53 | missense | c.844C>G(p.R282G) |
| P01 | CYLD | splice_site | c.1949+1G>A |
| P01 | KMT2B | missense | c.3292G>A(p.G1098R) |
| P01 | FGF19 | Amplification |  |
| P02 | FBXW7 | missense | c.463C>A(p.H155N) |
| P02 | PIK3R2 | stop_gained | c.1576G>T(p.E526*) |
| P02 | KRAS | missense | c.34G>T(p.G12C) |
| P02 | ALK | missense | c.3498G>A(p.M1166I) |
| P02 | FGFR3 | missense | c.670G>T(p.G224C) |
| P02 | NTRK1 | missense | c.2230C>A(p.R744S) |
| P02 | CDKN2A | stop_gained | c.216C>A(p.C72*) |
| P02 | TP53 | missense | c.463A>C(p.T155P) |
| P02 | RICTOR | splice_site | c.4137-1G>A |
| P02 | DAXX | missense | c.863A>T(p.D288V) |
| P02 | GRM3 | missense | c.709C>A(p.R237S) |
| P02 | PAK3 | missense | c.339G>T(p.K113N) |
| P02 | PKHD1 | missense | c.11542G>T(p.V3848F) |
| P02 | POLE | missense | c.6116G>T(p.G2039V) |
| P02 | MYD88 | missense | c.490G>T(p.D164Y) |
| P03 | TSC2 | splice_site | c.649-2A>G |
| P03 | TP53 | missense | c.646G>A(p.V216M) |
| P03 | DNMT3A | missense | c.2407A>G(p.R803G) |
| P04 | TP53 | missense | c.841G>C(p.D281H) |
| P04 | ATRX | missense | c.659G>T(p.C220F) |
| P04 | NPM1 | missense | c.559G>A(p.E187K) |
| P04 | NQO1 | missense | c.607C>G(p.Q203E) |
| P04 | PTPRD | Deletion |  |
| P05 | YAP1 | Amplification |  |
| P05 | PIK3CA | missense | c.1633G>A(p.E545K) |
| P05 | ATR | missense | c.7817G>A(p.R2606Q) |
| P05 | NSD1 | missense | c.1000C>T(p.R334C) |
| P05 | PRF1 | missense | c.208G>A(p.D70N) |
| P05 | TTF1 | missense | c.2680T>C(p.S894P) |
| P05 | POLD1 | missense | c.283A>G(p.I95V) |
| P05 | VHL | Deletion |  |
| P06 | HRAS | missense | c.34G>A(p.G12S) |
| P06 | MET | missense | c.633G>C(p.L211F) |
| P06 | SMAD4 | missense | c.730C>A(p.P244T) |
| P06 | TP53 | stop_gained | c.574C>T(p.Q192*) |
| P06 | FANCC | stop_gained | c.994C>T(p.Q332*) |
| P06 | PKD1 | missense | c.4474C>T(p.R1492C) |
| P06 | MCL1 | Amplification |  |
| P07 | CCND1 | Amplification |  |
| P07 | BRAF | splice_site | c.2128-4_2133delGTAGATTCTCinsTTTTTTTTTTTGAGATGGAGTTTTTCTCTTCTT |
| P07 | IGF1R | missense | c.3086G>C(p.R1029T) |
| P07 | TP53 | indel | c.569_577delCTCCTCAGC(p.P190_Q192del) |
| P07 | CDKN2A | Deletion |  |
| P07 | AMER1 | Deletion |  |
| P07 | TET2 | stop_gained | c.268C>T(p.Q90*) |
| P07 | ATR | missense | c.1948G>C(p.E650Q) |
| P07 | BAP1 | stop_gained | c.1306C>T(p.Q436*) |
| P07 | ARID2 | frameshift | c.3942_3943delTG(p.S1314Rfs*3) |
| P07 | FGF19 | Amplification |  |
| P07 | PTPRD | Deletion |  |
| P08 | CDKN2B | Deletion |  |
| P08 | MYC | Amplification |  |
| P08 | EP300 | missense | c.4241A>G(p.Y1414C) |
| P08 | TP53 | missense | c.646G>A(p.V216M) |
| P08 | CDKN2A | Deletion |  |
| P08 | DNMT3A | splice_site | c.2408+2T>G |
| P08 | AXL | missense | c.790C>A(p.L264M) |
| P08 | GRM3 | missense | c.1352A>G(p.D451G) |
| P08 | MLH3 | missense | c.2606C>T(p.S869L) |
| P08 | SMARCA4 | missense | c.1609G>A(p.G537R) |
| P08 | TET2 | missense | c.822C>G(p.I274M) |
| P08 | PHOX2B | missense | c.254T>C(p.L85P) |
| P08 | PTK2 | Amplification |  |
| P08 | ZNF217 | Amplification |  |
| P08 | TTF1 | Amplification |  |
| P08 | PTPRD | Deletion |  |
| P09 | CCNE1 | Amplification |  |
| P09 | MYC | Amplification |  |
| P09 | KDM5A | Amplification |  |
| P09 | NOTCH1 | Deletion |  |
| P09 | TSC1 | Deletion |  |
| P09 | AKT2 | Amplification |  |
| P09 | NRAS | Amplification |  |
| P09 | KRAS | Amplification |  |
| P09 | TP53 | stop_gained | c.592G>T(p.E198*) |
| P09 | MDM2 | Amplification |  |
| P09 | PKD1 | missense | c.5421C>G(p.I1807M) |
| P09 | BMPR1A | Deletion |  |
| P09 | AXL | Amplification |  |
| P09 | TOP1 | Amplification |  |
| P09 | SRC | Amplification |  |
| P10 | CCND1 | Amplification |  |
| P10 | MYC | Amplification |  |
| P10 | BRAF | missense | c.1798G>T(p.V600L) |
| P10 | KRAS | missense | c.35G>T(p.G12V) |
| P10 | JAK2 | Amplification |  |
| P10 | TP53 | missense | c.838A>G(p.R280G) |
| P10 | AMER1 | missense | c.1417G>T(p.D473Y) |
| P10 | FLT4 | splice_site | c.2761+44_2850+18delinsA |
| P10 | AR | missense | c.1711C>T(p.H571Y) |
| P10 | BRIP1 | missense | c.2285G>T(p.R762L) |
| P10 | IDH1 | missense | c.395G>T(p.R132L) |
| P10 | KDR | missense | c.1133A>T(p.K378I) |
| P10 | FGF19 | Amplification |  |
| P10 | ZNF703 | Amplification |  |
| P10 | CD274 | Amplification |  |
| P10 | PTK2 | Amplification |  |
| P10 | RECQL4 | Amplification |  |
| P10 | PDCD1LG2 | Amplification |  |
| P11 | KEAP1 | missense | c.1244G>T(p.R415L) |
| P11 | PTEN | missense | c.425G>T(p.R142L) |
| P11 | AKT3 | missense | c.740G>C(p.R247P) |
| P11 | PIK3C3 | missense | c.22C>T(p.H8Y) |
| P11 | ERBB4 | missense | c.2777C>T(p.T926M) |
| P11 | NF1 | stop_gained | c.7738G>T(p.E2580*) |
| P11 | NTRK3 | missense | c.1195C>T(p.P399S) |
| P11 | RET | missense | c.740C>A(p.A247D) |
| P11 | ROS1 | missense | c.1970C>T(p.P657L) |
| P11 | SMAD4 | missense | c.1094G>T(p.G365V) |
| P11 | TP53 | splice_region | c.375G>T(p.T125=) |
| P11 | ATM | stop_gained | c.7843C>T(p.Q2615*) |
| P11 | FH | splice_region | c.135A>T(p.A45=) |
| P11 | DNMT3A | missense | c.2330C>G(p.P777R) |
| P11 | PDE11A | splice_site | c.162+1G>A |
| P11 | CHD4 | missense | c.1953G>T(p.Q651H) |
| P11 | LZTR1 | stop_gained | c.115G>T(p.E39*) |
| P11 | ABCB1 | missense | c.1483G>T(p.V495F) |
| P11 | AR | missense | c.1147C>T(p.P383S) |
| P11 | ARID1A | stop_gained | c.3871G>T(p.E1291*) |
| P11 | ARID1A | missense | c.5992G>T(p.D1998Y) |
| P11 | ATR | frameshift | c.330dupT(p.A111Cfs*15) |
| P11 | BAI3 | missense | c.92C>T(p.S31L) |
| P11 | BRCA2 | missense | c.1312G>C(p.D438H) |
| P11 | DICER1 | stop_gained | c.5026A>T(p.R1676*) |
| P11 | FANCL | missense | c.1018G>A(p.E340K) |
| P11 | FANCM | stop_gained | c.2671C>T(p.Q891*) |
| P11 | GATA3 | missense | c.403C>A(p.P135T) |
| P11 | GNAS | missense | c.1318G>A(p.D440N) |
| P11 | GRM8 | missense | c.914G>T(p.W305L) |
| P11 | HGF | frameshift | c.781delC(p.R261Afs*33) |
| P11 | JAK1 | missense | c.2843G>C(p.G948A) |
| P11 | LHCGR | missense | c.1387G>A(p.E463K) |
| P11 | MSH6 | missense | c.2178C>A(p.F726L) |
| P11 | PAX5 | missense | c.1151C>A(p.A384D) |
| P11 | PLK1 | missense | c.1535G>C(p.R512P) |
| P11 | PTCH1 | missense | c.1133G>T(p.G378V) |
| P11 | SDHA | stop_gained | c.982G>T(p.E328*) |
| P12 | FAT1 | missense | c.11253G>T(p.K3751N) |
| P12 | MYCN | missense | c.799G>T(p.D267Y) |
| P12 | CREBBP | missense | c.740C>G(p.S247C) |
| P12 | PIK3CA | missense | c.1633G>A(p.E545K) |
| P12 | RICTOR | missense | c.4721C>T(p.S1574F) |
| P12 | MET | intron | c.1201-10545_1448dup(p.H484Ffs*7) |
| P12 | ALK | missense | c.3586C>A(p.L1196M) |
| P12 | MAP2K1 | missense | c.958G>C(p.E320Q) |
| P12 | SMAD2 | missense | c.878C>T(p.S293L) |
| P12 | TP53 | missense | c.733G>A(p.G245S) |
| P12 | ATM | missense | c.5494G>A(p.E1832K) |
| P12 | HNF1A | frameshift | c.872dupC(p.G292Rfs*25) |
| P12 | BAP1 | frameshift | c.527dupT(p.T177Hfs*6) |
| P12 | NSD1 | missense | c.152C>G(p.S51W) |
| P12 | PTCH1 | missense | c.1120G>A(p.E374K) |
| P12 | SETBP1 | stop_gained | c.3202C>T(p.Q1068*) |
| P12 | STAT3 | missense | c.346C>G(p.L116V) |
| P12 | TERT | missense | c.58G>A(p.E20K) |
| P12 | TERT | Amplification |  |
| P13 | FAT1 | missense | c.7528C>G(p.L2510V) |
| P13 | MYC | Amplification |  |
| P13 | NOTCH1 | frameshift | c.4657delG(p.E1553Sfs*27) |
| P13 | PIK3R1 | indel | c.1699_1701delAAA(p.K567del) |
| P13 | PIK3CA | Amplification |  |
| P13 | ALK | Amplification |  |
| P13 | TP53 | frameshift | c.454_455delCCinsT(p.P152Cfs*18) |
| P13 | TP53 | missense | c.454C>T(p.P152S) |
| P13 | DNMT3A | frameshift | c.1874delC(p.P625Lfs*26) |
| P13 | PBRM1 | stop_gained | c.1600C>T(p.R534*) |
| P13 | PKHD1 | missense | c.218G>A(p.R73Q) |
| P13 | TNFAIP3 | missense | c.2290G>T(p.A764S) |
| P13 | TERC | Amplification |  |
| P13 | SOX2 | Amplification |  |
| P13 | MECOM | Amplification |  |
| P13 | TERT | Amplification |  |
| P13 | IL7R | Amplification |  |
| P14 | CDKN2B | Deletion |  |
| P14 | NF2 | frameshift | c.1349delA(p.E450Gfs*5) |
| P14 | MYC | Amplification |  |
| P14 | RICTOR | Amplification |  |
| P14 | TP53 | missense | c.844C>T(p.R282W) |
| P14 | CDKN2A | Deletion |  |
| P14 | PTK2 | Amplification |  |
| P14 | RECQL4 | Amplification |  |
| P14 | IL7R | Amplification |  |
| P15 | KDM5A | missense | c.599A>C(p.D200A) |
| P15 | MTOR | indel | c.5516_5527delCCACCACTGCCA(p.T1839_A1842del) |
| P15 | FLT3 | missense | c.2858C>T(p.A953V) |
| P15 | ARID1A | missense | c.211A>G(p.K71E) |
| P15 | ATRX | missense | c.514A>T(p.T172S) |
| P15 | BRCA2 | missense | c.2702T>G(p.L901R) |
| P15 | BUB1B | missense | c.1646C>T(p.P549L) |
| P15 | C11orf30 | missense | c.1189C>A(p.L397I) |
| P15 | FANCE | missense | c.331G>A(p.E111K) |
| P15 | GATA2 | missense | c.188C>T(p.P63L) |
| P15 | IL7R | missense | c.487T>G(p.L163V) |
| P15 | JUN | missense | c.754C>T(p.R252W) |
| P15 | MED12 | indel | c.6285_6287delACA(p.Q2115del) |
| P15 | MUTYH | missense | c.1538C>T(p.P513L) |
| P15 | NSD1 | missense | c.6341C>T(p.T2114I) |
| P15 | PMS2 | missense | c.1382C>T(p.A461V) |
| P15 | SDHB | indel | c.478_480delAAG(p.K160del) |
| P15 | SETBP1 | missense | c.2415G>A(p.M805I) |
| P15 | TEK | missense | c.149G>A(p.R50H) |
| P16 | INPP4B | splice_site | c.1564-2A>G |
| P16 | PTEN | missense | c.127G>C(p.E43Q) |
| P16 | KRAS | missense | c.34G>T(p.G12C) |
| P16 | ERBB4 | missense | c.1883C>A(p.P628H) |
| P16 | TP53 | missense | c.614A>G(p.Y205C) |
| P16 | TOP2A | splice_site | c.4132+1G>T |
| P16 | ABCB1 | missense | c.3784G>T(p.A1262S) |
| P16 | MED12 | missense | c.1445A>G(p.N482S) |
| P16 | ARID2 | missense | c.1372G>T(p.G458C) |
| P16 | DPYD | missense | c.458G>T(p.G153V) |
| P16 | GNAS | missense | c.1606C>G(p.Q536E) |
| P16 | IDH1 | missense | c.594A>T(p.Q198H) |
| P16 | MAP3K1 | missense | c.878C>T(p.P293L) |
| P16 | PRKCI | missense | c.1232G>A(p.S411N) |
| P16 | STAG2 | missense | c.1256C>T(p.S419L) |
| P17 | FBXW7 | missense | c.1436G>A(p.R479Q) |
| P17 | KRAS | missense | c.38G>A(p.G13D) |
| P17 | ATM | missense | c.1009C>T(p.R337C) |
| P17 | APC | stop_gained | c.712C>T(p.Q238*) |
| P17 | ARID1A | missense | c.67G>A(p.E23K) |
| P17 | ARID2 | frameshift | c.1097_1100delGGGA(p.R366Ifs*4) |
| P17 | KMT2B | missense | c.4013G>T(p.C1338F) |
| P18 | KRAS | missense | c.35G>T(p.G12V) |
| P18 | FGFR2 | stop_gained | c.870G>A(p.W290*) |
| P18 | SMAD4 | stop_gained | c.97G>T(p.E33*) |
| P18 | SMAD4 | missense | c.353C>T(p.A118V) |
| P18 | CDKN2A | stop_gained | c.131dupA(p.Y44*) |
| P18 | TP53 | missense | c.843C>A(p.D281E) |
| P18 | GRM3 | missense | c.709C>T(p.R237C) |
| P18 | HGF | Amplification |  |
| P19 | TSC1 | splice_site | c.664-1G>A |
| P19 | STK11 | Deletion |  |
| P19 | PIK3R2 | Amplification |  |
| P19 | RPTOR | Amplification |  |
| P19 | RICTOR | Amplification |  |
| P19 | FGFR2 | missense | c.1647T>A(p.N549K) |
| P19 | FLT3 | missense | c.927G>T(p.M309I) |
| P19 | PDGFRA | missense | c.848A>G(p.K283R) |
| P19 | RET | missense | c.2653G>C(p.G885R) |
| P19 | SMAD4 | Deletion |  |
| P19 | TP53 | stop_gained | c.892G>T(p.E298*) |
| P19 | CDKN2A | missense | c.383G>C(p.R128P) |
| P19 | CDKN2A | frameshift | c.458delA(p.D153Afs*40) |
| P19 | TP53 | missense | c.844C>T(p.R282W) |
| P19 | KDR | splice_site | c.3511-1G>T |
| P19 | MRE11A | splice_site | c.1564-2A>T |
| P19 | BAP1 | missense | c.569C>T(p.P190L) |
| P19 | EPHA5 | missense | c.1264A>G(p.K422E) |
| P19 | EXT1 | missense | c.1714A>G(p.T572A) |
| P19 | FANCA | missense | c.1728G>T(p.R576S) |
| P19 | KDR | missense | c.191C>A(p.P64H) |
| P19 | LYN | stop_gained | c.1168A>T(p.R390*) |
| P19 | MAP3K1 | missense | c.528G>C(p.M176I) |
| P19 | MLH3 | missense | c.2962A>G(p.I988V) |
| P19 | MRE11A | missense | c.1701C>G(p.N567K) |
| P19 | MSH6 | missense | c.1615C>G(p.L539V) |
| P19 | SMARCB1 | missense | c.1127G>C(p.R376T) |
| P19 | ZNF703 | missense | c.856G>T(p.A286S) |
| P19 | KMT2B | missense | c.1126A>G(p.K376E) |
| P19 | TOP1 | Amplification |  |
| P19 | GNAS | Amplification |  |
| P19 | ZNF217 | Amplification |  |
| P19 | TERT | Amplification |  |
| P19 | SRC | Amplification |  |
| P19 | IL7R | Amplification |  |
| P20 | AKT1 | missense | c.1216C>A(p.R406S) |
| P20 | PIK3R1 | indel | c.1644_1673dupCTTGAAGAAGCAGGCAGCTGAGTATCGAGA(p.D548_R557dup) |
| P20 | KRAS | Amplification |  |
| P20 | CDKN2A | frameshift | c.40dupG(p.D14Gfs*30) |
| P20 | TP53 | missense | c.734G>A(p.G245D) |
| P20 | FANCD2 | stop_gained | c.3399_3457delinsCCTCTACTAAAAAAAAAAAAAAAAAA(p.I1135_E1153delinsYXKKKKKK) |
| P20 | EPHA5 | missense | c.1326G>T(p.E442D) |
| P20 | GRM8 | missense | c.1962C>G(p.F654L) |
| P20 | KMT2B | missense | c.860G>A(p.R287H) |
| P20 | MECOM | missense | c.755G>C(p.G252A) |
| P20 | ZNF217 | Amplification |  |
| P20 | ZNF703 | Amplification |  |
| P20 | GNAS | Amplification |  |
| P21 | NOTCH2 | missense | c.5398C>T(p.R1800C) |
| P21 | RAC1 | missense | c.404C>T(p.T135I) |
| P21 | CTCF | stop_gained | c.1492_1493ins(19033)(p.E507VfsX40) |
| P21 | DICER1 | missense | c.3827A>T(p.D1276V) |
| P21 | RHOA | missense | c.524C>T(p.T175M) |
| P21 | TSHR | missense | c.1802A>G(p.Y601C) |
| P21 | DDR2 | missense | c.2141G>A(p.R714Q) |
| P21 | HNF1A | missense | c.947A>G(p.K316R) |
| P21 | KDR | missense | c.3291A>C(p.E1097D) |
| P22 | SMAD4 | missense | c.1088G>A(p.C363Y) |
| P22 | ATM | stop_gained | c.5130G>A(p.W1710*) |
| P22 | ATM | missense | c.6518T>G(p.L2173R) |
| P22 | PRSS1 | splice_region | c.453C>T(p.G151=) |
| P22 | ESR1 | missense | c.721C>T(p.R241W) |
| P22 | GNAS | missense | c.601C>T(p.R201C) |
| P22 | PTPRD | Deletion |  |
| P23 | RPTOR | missense | c.3400G>C(p.E1134Q) |
| P23 | KRAS | missense | c.34_35delGGinsTT(p.G12F) |
| P23 | NTRK1 | missense | c.476T>A(p.L159Q) |
| P23 | NTRK3 | missense | c.1938G>T(p.Q646H) |
| P23 | ERBB4 | missense | c.3909C>A(p.H1303Q) |
| P23 | SMAD4 | stop_gained | c.538C>T(p.Q180*) |
| P23 | TP53 | stop_gained | c.892G>T(p.E298*) |
| P23 | ARID2 | stop_gained | c.4252G>T(p.G1418*) |
| P23 | CTLA4 | missense | c.230C>G(p.A77G) |
| P23 | FANCD2 | missense | c.932T>G(p.V311G) |
| P23 | FGF19 | missense | c.316G>T(p.D106Y) |
| P23 | GRM8 | missense | c.2204A>T(p.K735M) |
| P23 | IGF2 | missense | c.199G>T(p.V67F) |
| P23 | IL7R | stop_gained | c.704C>A(p.S235*) |
| P23 | RELN | missense | c.5263G>A(p.D1755N) |
| P23 | STAG2 | stop_gained | c.142A>T(p.K48*) |
| P23 | C11orf30 | missense | c.2126G>C(p.G709A) |
| P23 | DENND1A | stop_gained | c.2381C>G(p.S794*) |
| P23 | DENND1A | frameshift | c.3016delG(p.E1006Rfs*59) |
| P23 | GNAS | indel | c.1359_1394delCGACTCCGGGGCGGCCCCTGACGCCCCAGCCGATCC(p.S455_D466del) |
| P23 | GNAS | missense | c.1362_1363delCTinsTG(p.S455A) |
| P23 | MRE11A | missense | c.1025A>T(p.E342V) |
| P23 | RECQL4 | frameshift | c.610delG(p.A204Pfs*9) |
| P23 | PTPRD | Deletion |  |
| P23 | SOX2 | Amplification |  |
| P23 | TERC | Amplification |  |
| P23 | GNAS | Amplification |  |
| P23 | TOP2A | Amplification |  |
| P23 | MCL1 | Amplification |  |
| P23 | ZNF217 | Amplification |  |
| P24 | ERBB2 | Amplification |  |
| P24 | TP53 | missense | c.644G>A(p.S215N) |
| P24 | CDKN2A | Deletion |  |
| P24 | TERT | upstream | c.-124C>T |
| P24 | SMO | indel | c.67_69delCTG(p.L23del) |
| P24 | CDK12 | Amplification |  |
| P25 | PIK3CA | missense | c.1624G>A(p.E542K) |
| P25 | KRAS | missense | c.35G>A(p.G12D) |
| P25 | RAC1 | indel | c.60_62delGAT(p.I21del) |
| P25 | SMAD4 | frameshift | c.901dupT(p.Y301Lfs*7) |
| P25 | TP53 | missense | c.473G>T(p.R158L) |
| P25 | RNF43 | stop_gained | c.115G>T(p.E39*) |
| P25 | ABCB1 | missense | c.2146T>C(p.F716L) |
| P25 | ARID2 | frameshift | c.4666delG(p.V1556Cfs*10) |
| P25 | BARD1 | missense | c.793T>G(p.S265A) |
| P25 | MAP3K1 | missense | c.2428T>C(p.S810P) |
| P25 | PRSS1 | indel | c.64_66delGAC(p.D22del) |
| P25 | TMPRSS2 | missense | c.145G>A(p.V49M) |
| P26 | FAT1 | missense | c.5255T>A(p.L1752Q) |
| P26 | MYC | Amplification |  |
| P26 | INPP4B | missense | c.550G>T(p.V184L) |
| P26 | KRAS | missense | c.34G>T(p.G12C) |
| P26 | KRAS | Amplification |  |
| P26 | TGFBR2 | missense | c.739G>T(p.D247Y) |
| P26 | ATM | missense | c.7219T>C(p.S2407P) |
| P26 | MDM2 | Amplification |  |
| P26 | DNMT3A | missense | c.2645G>C(p.R882P) |
| P26 | ARID1B | indel | c.670_933del(p.P224_G311del) |
| P26 | FANCA | missense | c.4081T>C(p.Y1361H) |
| P26 | LRP1B | missense | c.4097G>C(p.G1366A) |
| P26 | PRKCI | missense | c.1285G>A(p.D429N) |

**Supplementary Table 3. The LOH and HRR status of CUP patients.**

| **Patient ID** | **LOH** | **HRR** |
| --- | --- | --- |
| P01 | 0.276 | WT |
| P02 | 0.278 | WT |
| P03 | 0.161 | WT |
| P04 | 0.300 | WT |
| P05 | 0.448 | MUT |
| P06 | 0.112 | MUT |
| P07 | 0.259 | MUT |
| P08 | 0.381 | WT |
| P09 | 0.474 | WT |
| P10 | 0.446 | MUT |
| P11 | 0.189 | MUT |
| P12 | 0.380 | MUT |
| P13 | 0.283 | WT |
| P14 | 0.334 | WT |
| P15 | 0.090 | MUT |
| P16 | 0.349 | MUT |
| P17 | 0.370 | MUT |
| P18 | 0.252 | WT |
| P19 | 0.268 | MUT |
| P20 | 0.206 | MUT |
| P21 | 0.118 | WT |
| P22 | 0.188 | MUT |
| P23 | 0.337 | MUT |
| P24 | 0.382 | WT |
| P25 | 0.308 | MUT |
| P26 | 0.149 | MUT |
